# Antenatal sildenafil citrate treatment increases offspring blood pressure in the placental-specific *Igf2* knockout mouse model of FGR

**DOI:** 10.1152/ajpheart.00568.2019

**Published:** 2019-12-06

**Authors:** L. J. Renshall, E. C. Cottrell, E. Cowley, C. P. Sibley, P. N. Baker, E. B. Thorstensen, S. L. Greenwood, M. Wareing, M. R. Dilworth

**Affiliations:** ^1^Maternal and Fetal Health Research Centre, Division of Developmental Biology and Medicine, School of Medicine, Faculty of Biology, Medicine and Health, The University of Manchester, Manchester, United Kingdom; ^2^Manchester Academic Health Science Centre, Central Manchester University Hospitals NHS Foundation Trust, St. Mary's Hospital, Manchester, United Kingdom; ^3^Liggins Institute, The University of Auckland, Grafton, Auckland, New Zealand; ^4^College of Life Sciences, University of Leicester, Leicester, United Kingdom

**Keywords:** blood pressure, fetal growth restriction, offspring, sildenafil citrate

## Abstract

Fetal growth restriction (FGR), where a fetus fails to reach its genetic growth potential, affects up to 8% of pregnancies and is a major risk factor for stillbirth and adulthood morbidity. There are currently no treatments for FGR, but candidate therapies include the phosphodiesterase-5 inhibitor sildenafil citrate (SC). Randomized clinical trials in women demonstrated no effect of SC on fetal growth in cases of severe early onset FGR; however, long-term health outcomes on the offspring are unknown. This study aimed to assess the effect of antenatal SC treatment on metabolic and cardiovascular health in offspring by assessing postnatal weight gain, glucose tolerance, systolic blood pressure, and resistance artery function in a mouse model of FGR, the placental-specific insulin-like growth factor 2 (PO) knockout mouse. SC was administered subcutaneously (10 mg/kg) daily from embryonic day (E)12.5. Antenatal SC treatment did not alter fetal weight or viability but increased postnatal weight gain in wild-type (WT) female offspring (*P* < 0.05) and reduced glucose sensitivity in both WT (*P* < 0.01) and P0 (*P* < 0.05) female offspring compared with controls. Antenatal SC treatment increased systolic blood pressure in both male (WT vs. WT-SC: 117 ± 2 vs. 140 ± 3 mmHg, *P* < 0.0001; P0 vs. P0-SC: 113 ± 3 vs. 140 ± 4 mmHg, *P* < 0.0001; means ± SE) and female (WT vs. WT-SC: 121 ± 2 vs. 140 ± 2 mmHg, *P* < 0.0001; P0 vs. P0-SC: 117 ± 2 vs. 144 ± 4 mmHg, *P* < 0.0001) offspring at 8 and 13 wk of age. Increased systolic blood pressure was not attributed to altered mesenteric artery function. In utero exposure to SC may result in metabolic dysfunction and elevated blood pressure in later life.

**NEW & NOTEWORTHY** Sildenafil citrate (SC) is currently used to treat fetal growth restriction (FGR). We demonstrate that SC is ineffective at treating FGR, and leads to a substantial increase systolic blood pressure and alterations in glucose homeostasis in offspring. We therefore urge caution and suggest that further studies are required to assess the safety and efficacy of SC in utero, in addition to the implications on long-term health.

## INTRODUCTION

Fetal growth restriction (FGR), the inability of a fetus to reach its predetermined genetic growth potential, is a major complication of pregnancy affecting up to 8% of births in the UK (13). FGR infants are at increased risk of mortality and morbidity both in the short and longer term (2, 25). FGR fetuses are 10 times more likely to be stillborn than appropriately grown fetuses (1, 4, 15). There are no current therapies for FGR, in part due to the possible teratogenic effects of pharmaceutical agents (48). This risk has led to a reluctance within the pharmaceutical industry to develop drugs for obstetric complications and the repurposing of therapies currently used in nonpregnant individuals (8, 14, 39). In the absence of treatments, in cases of severe early onset FGR, the only clinical option is premature delivery of the baby, which, in itself, increases the risk of adverse effects on maternal and fetal health (22) and is associated with increased morbidity in adulthood (9, 21).

One drug that has been repurposed as a potential therapeutic for FGR is the potent vasodilator sildenafil citrate (SC). SC (marketed as Viagra) is a selective phosphodiesterase type 5 (PDE-5) inhibitor that inhibits the hydrolysis of cyclic guanosine monophosphate (cGMP), thus prolonging the actions of the vasodilator nitric oxide (NO). SC dilated myometrial arteries of women with FGR ex vivo but was without effect on arteries from normal pregnancies (47). In preclinical studies, SC treatment increased fetal weight in the catechol-*O*-methyltransferase knockout (*COMT*^−/−^) mouse model of FGR by improving aberrant umbilical artery blood flow (43). We (10) also demonstrated that SC, administered via drinking water, increased the weight of FGR fetuses of the placental-specific insulin-like growth factor 2 (*Igf2* P0^+/−^) knockout mouse via an overall increase in nutrient transfer capacity of the placenta. In two separate studies, SC treatment in growth-restricted ovine fetuses also led to an increase in fetal weight as a result of an increase in nutrient transfer capacity of the placenta (30, 38). However, in the single umbilical artery ligation (SUAL) sheep model of FGR, SC led to reduced uterine blood flow as well as reduced Po_2_, hypotension, and tachycardia in fetuses from both normal and SUAL ewes (27). Overall, these data suggest that the underlying etiology of FGR may determine whether SC is beneficial.

Following these preclinical studies, and a small nonrandomized clinical trial suggesting that maternal SC may increase fetal abdominal growth velocity (45), the multicenter randomized control trial “Sildenafil Therapy In Dismal Prognosis Severe Early Onset IUGR” (STRIDER) commenced. Despite the wealth of preclinical data suggesting effectiveness of SC at increasing fetal growth, the clinical trial found that SC, compared with placebo, did not prolong pregnancy, or have any effect on fetal growth velocity or fetal or neonatal survival rates (18, 41). Furthermore, the Dutch STRIDER trial was halted, as there was an increased incidence of lung complications in babies from mothers who had taken SC during pregnancy (19). The question of whether antenatal treatment with SC resulted in long-term health implications for the offspring remains unanswered following these trials. However, recent preclinical data demonstrated that treating endothelial nitric oxide synthase knockout (*eNOS*^−/−^) mice with SC during pregnancy resulted in a significant rise in systolic blood pressure (SBP), which was associated with a constrictive phenotype in mesenteric arteries (28). In the same study, C57BL/6J offspring from dams exposed to SC during pregnancy showed increased endothelial-dependent relaxation in isolated mesenteric arteries with no change in SBP; these data suggest that *eNOS*^−/−^ mice may be more vulnerable to negative effects associated with SC. We therefore chose to assess the long-term implications of antenatal SC in the *Igf2* P0^+/−^ (P0) knockout mouse, as this model of FGR is not characterized by a cardiovascular phenotype but does show evidence of altered placental morphology and function akin to human FGR (7, 12, 42).

For this study, we sought to reproduce the concentration of SC in maternal blood from previous (35) and recently completed clinical trials (18, 41). Pregnant dams were therefore given a subcutaneous injection of 10 mg/kg SC or saline. Postnatal weight gain, glucose tolerance, blood pressure, and resistance artery function in adult male and female offspring of both wild-type (WT) and P0 genotypes were then assessed. We hypothesized that maternal SC treatment of the P0 knockout mouse would have no detrimental effects on cardiovascular function of the offspring.

## METHODS

### Ethical Approval

All procedures were performed in accordance with the UK Animals Scientific Procedures Act (1986) and under the provision of a UK Home Office project license (PPL 40/3385 and P9755892D). Work was approved by the local animal welfare and ethical review board of the University of Manchester. This study is reported according to the ARRIVE guidelines (23). Mice were fed a standard pellet chow (BK001 diet, Special Dietary Services) with ad libitum access to water (Hydropac, Laboratory Products) and were caged in individually ventilated cages under a 12-h:12-h light-dark cycle at 21–23°C with 65% humidity.

### P0^+/−^ Mouse

Males (12–26 wk old) heterozygous for the deletion of the P0 transcript were mated with 8- to 12-wk-old virgin C57BL/6J (WT) females. A total of 76 females were mated, with 48 confirmed pregnancies. Identification of a vaginal plug the following morning was deemed to be the beginning of pregnancy and designated embryonic *day 0.5* (E0.5; term ~E19.5). Litters contained both WT and P0 (growth-restricted) fetuses. Mice were originally a kind gift from Wolf Reik and Miguel Constância (6).

### SC Treatment

**Fig. 1.**
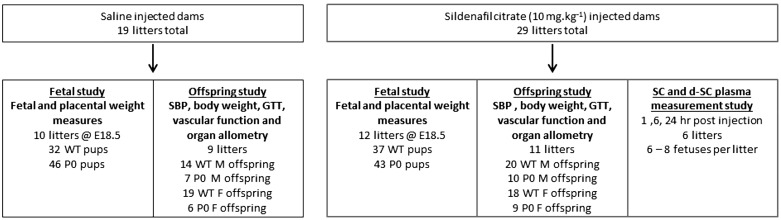
Study design. Number of litters and pups [wild type (WT) and placental-specific insulin-like growth factor 2 knockout (P0)] in each of the treatment groups are shown. For each treatment group, litters were separated into 2 study arms. The fetal study arm assessed effects of maternal sildenafil citrate (SC) treatment on fetal weight at embryonic day (E)18.5, whereas the offspring study arm assessed effects of antenatal SC treatment on postnatal body weight, glucose tolerance, systolic blood pressure (SBP), resistance artery function, and organ allometry. Terminal blood samples, used for measuring SC and desmethylsildenafil (d-SC), were taken between E17.5 and E18.5 from dams/fetuses treated with SC.

To assess the effects of antenatal SC treatment on fetal and placental weight, litter size, and viability, dams (the experimental unit) were randomly assigned to receive either a subcutaneous injection in the skinfold between the scapulae of 10 mg/kg sc (Pfizer, UK, *n* = 10 dams) or 0.9% saline (*n* = 12 dams; control) daily (9:00–10:00 AM) from E12.5 up to and including E17.5 (see Fig. 1 for study design). Maternal body weight at E18.5 was similar between saline and SC groups (38.8 ± 1.4 and 38.4 ± 1.0 g, respectively; means ± SE). Saline and SC treatment regimens were performed in parallel. We determined the animal equivalent dose [AED (29)] in mouse based on the amount of SC received by women recruited into the STRIDER clinical trial (25 mg 3 times/day, i.e., a total of 75 mg/day). The AED approximated to 13.2 mg/kg based on a pregnant woman with a weight of 70 kg, but this was reduced to 10 mg/kg in mouse to compare with similar studies in rat (36). We employed a daily subcutaneous injection dosing regimen, thus ensuring that mice received an exact dose of SC as opposed to previous treatments in drinking water (10, 28, 43). A separate set of experiments were performed on offspring (the experimental unit) from dams that were administered either SC (*n* = 11 dams) or saline (*n* = 9 dams) as above but then were allowed to litter down. We chose to assess outcomes from all pups from each of the litters for two reasons: *1*) we were unable to reliably identify WT and P0 genotype until ear clipping at 4 wk of age; and *2*) there were consistently lower survival rates of P0 offspring (between birth and 4 wk of age); thus, to increase the likelihood of at least one P0 offspring being present within a litter, all male and female offspring were used in all experiments.

### Measurement of Maternal and Fetal Plasma SC Concentration

In a separate set of experiments 10- to 13-wk-old dams (*n* = 3) were euthanized by cervical dislocation 1 h (*n* = 1 dam) or 24 h (*n* = 2 dams) after a single injection of 10 mg/kg SC at E17.5. Following decapitation, trunk blood samples were collected in lithium-heparinized capillary tubes (Sarstedt). Blood from all fetuses (WT and P0 genotypes and both sexes) from each of the dams was also pooled for fetal measurements (1-h time point, 1 litter; 24-h time point, 2 litters). Blood was centrifuged at 1,900 *g* for 5 min, and plasma was collected and stored at −80°C. SC and its breakdown metabolite desmethylsildenafil (d-SC) were measured using triple-quadrupole mass spectrometry utilizing previously described methods (49), with minor modifications. Briefly 20 µL of internal standard solution (200 ng/mL SC-d3 in water) and 20 µL of 0.02 M aqueous NaOH was added to 100 µL of plasma and mixed. The analytes were extracted using 1 mL of ethyl acetate (Merck, Darnstadt, Germany). After removal of the organic supernatant to a clean tube, samples were dried by vacuum concentration (Savant SC250EXP; Thermo Scientific, Asheville, NC). The dried extracts were resuspended in 70 µL of methanol-water-acetic acid solution (40:59:1 vol/vol/vol) and transferred to HPLC vials. Eight microliters was injected onto an HPLC mass spectrometer system consisting of an Accela MS pump and autosampler followed by a heated electrospray (HESI) source on a Finnigan TSQ Quantum Ultra AM triple-quadrupole mass spectrometer, all controlled by Finnigan Xcalibur software (Thermo Electron, San Jose, CA). The mobile phase was a gradient of methanol and water, both containing 0.1% formic acid, flowing at 250 µL/min through a Luna HST 2.6-µm C_18_ 100 × 3.0 mm column at 30°C (Phenomenex, Auckland, NZ). All compounds were eluted at 2.8 min. Ionization was in positive mode, and Q2 had 1.2 mTorr of argon for all target compounds. The mass transitions followed were: SC 475.1 → 283.0, SC-d3 478.1 → 283.0, and d-SC 461.1 → 283.0, all at 35 V. Unknowns were quantified against standards (1–1,000 ng/mL) run at the beginning of the assay. Quality control samples showed mean percent coefficients of variation of 3.5 and 3.8 for SC and d-SC, respectively.

### Fetal and Placental Weights

Dams were euthanized by cervical dislocation at E18.5 (i.e., 24 h after final treatment at E17.5), and a laparotomy was performed to remove fetuses and placentas from the uterine horns; fetal and placental wet weights were recorded.

### Fetal Weight Frequency Distribution Curves

Fetal weight histograms were formulated for individual fetal weights, as previously described (11). The fifth percentile of fetal weight was calculated as: (−Z critical value × standard deviation) + mean, where Z critical value (for the 5th centile from a Gaussian distribution) = 1.645.

### Genotyping

Using a previously described PCR method (10) fetal tail and offspring ear notch tissues were used to determine genotype of fetuses at E18.5 and offspring at *week 4* of age, respectively. Briefly, DNA was extracted from tissue (DNeasy, Qiagen) and P0^+/−^ mutants were identified using a three-primer system. A 740-bp mutant fragment contained within a 5-kb deletion site and a 495-bp WT fragment were amplified (mutant sense, 5′-TCCTGTACCTCCTAACTACCAC-3′, mutant antisense, 5′-GAGCCAGAAGCAAACT-3′, and WT sense 5′-CAATCTGCTCCTGCCTG-3′).

### Postnatal Body Weight

We attempted to standardize litters and weigh pups from birth. However, there were higher rates of mortality in P0 male and P0 female neonates than in WT fetuses, which was associated with maternal cannibalism. To avoid bias introduced by maternal interference, we chose to breed a minimum of nine litters for offspring studies. Offspring were caged with dams until weaning at 4 wk of age. At 4 wk of age, offspring were ear notched to aid identification of individual mice. A maximum of four female mice were housed in one cage. Male mice were separated and singly housed from 8 wk of age, as males were found to be aggressive toward littermates following separation for blood pressure measurements. Body weights were measured each week between *weeks 5* and *12*. Terminal body weights at *week 14* were also measured. The mean body weight at each age was calculated for individual groups.

### Glucose Tolerance Test

At 12 wk of age, mice were fasted overnight for 16 h with ad libitum access to water. A venepuncture was made at the base of the tail with a 25-gauge needle, and baseline blood glucose concentrations were measured using Accu-Chek Aviva (Roche Diagnostics) blood glucose monitoring system. Mice were then administered a 1 g/kg glucose (0.9% NaCl, 10% glucose) solution via intraperitoneal injection. Blood glucose concentrations at 15, 30, 60, 90, and 120 min postinjection were recorded.

### SBP Measurements in Offspring

SBP was measured at 8 and 13 wk of age (see Fig. 1 for study design) using a noninvasive blood pressure system (model LE5001, Panlab). Mice were acclimatized to the restraint tube to reduce stress before SBP measurements. Mice were transferred to a room with a temperature between 22 and 24°C on the day of measurement and left to acclimate for 30 min before measurements were recorded. First, a small rodent restraining tube was placed on a Thermopad heated mat (36°C, Harvard Apparatus); mice were not forced to enter the restraint tube, but when they entered they were secured with the tail exposed for SBP measurements. The tail cuff occlusion device and pulse transducer were placed on the tail for at least 10 min before recording of SBP. Heart rate was measured and did not exceed 700 beats/min. A total of 12 SBP measurements were recorded within 30 min and averaged (mean) for each individual mouse.

### Mesenteric Artery Function in Offspring

Second-order mesenteric arteries were harvested from offspring (14 wk) and mounted on a wire myograph (Danish Myo Technologies). Arteries were equilibrated and gassed with 20% O_2_-5% CO_2_, balance N_2_ and normalized to 0.9 of luminal pressure (L)_13.3 kPa_. Mesenteric artery contraction was assessed in response to depolarizing KPSS solution and U46619, (thromboxane A2 mimetic; 0.1–2,000 nM). U46619, cumulative dose response curves were constructed and used to determine EC_80_ values. Mesenteric arteries were then precontracted with an EC_80_ concentration of U46619, before endothelium-dependent [acetylcholine (ACh); 0.1–10,000 nM] and independent [sodium nitroprusside (SNP); 0.1–10,000 nM] relaxation was measured. Contraction was expressed as active effective pressure using the following equation: active effective pressure = (wall tension/2π)/vessel internal circumference. Relaxation was expressed as a percentage of EC_80_ precontraction to U46619.

### Offspring Organ Allometry

At 14 wk of age heart, kidney, spleen, lung, and brain were dissected from each mouse and weighed and presented as a percentage of body weight.

### Statistical Analyses

Studies were primarily powered to detect changes in fetal weight and offspring blood pressure. All studies were based on 80% statistical power at a 5% significance level.

#### Fetal and placental weight.

Data are presented as litter average (means ± SE) fetal or placental weight. Data were analyzed using two-way ANOVA with Bonferroni post hoc test.

#### Body weight.

The level of significant difference was determined using mean body weight for each group with repeated-measures two-way ANOVA and Sidak’s post hoc test.

#### Glucose tolerance.

Total area under the curve (AUC) values were calculated for each individual mouse. Mean AUC values for each group were calculated and used for statistical analyses. A Mann-Whitney *U*-test was used to analyze mean AUC for non-normally distributed data.

#### Blood pressure and mesenteric artery function.

Data are presented as means ± SE. Data were analyzed using two-way ANOVA with Bonferroni post hoc test.

#### Offspring organ allometry.

Data are presented as median [min–max]. To assess the effect of genotype and sex within a particular treatment group (i.e., saline or SC) a Kruskal-Wallis test was used. A Mann-Whitney *U*-test was used to compare the effect of SC treatment between two groups; e.g., WT female (F) from SC-treated pregnancy vs. WT F from saline-treated pregnancy.

## RESULTS

### Maternal and Fetal Plasma Measurements of SC and d-SC

In maternal plasma, SC and d-SC reached a peak of 693 and 27 ng/mL, respectively, 1 h postinjection (*n* = 1 dam) and declined to 26 [2–51] (SC) and 2 [1–3] (d-SC) ng/mL after 24 h (median [min−max], *n* = 2 dams). SC and d-SC reached 42 [26–59] and 6 [5–7] ng/mL in fetal plasma 1 h after injection (*n* = 1 litter) and SC and d-SC declined to 2 [2–2] and 1 [1–1] ng/mL after 24 h (*n* = 2 litters), respectively.

### Litter Characteristics

**Fig. 2.**
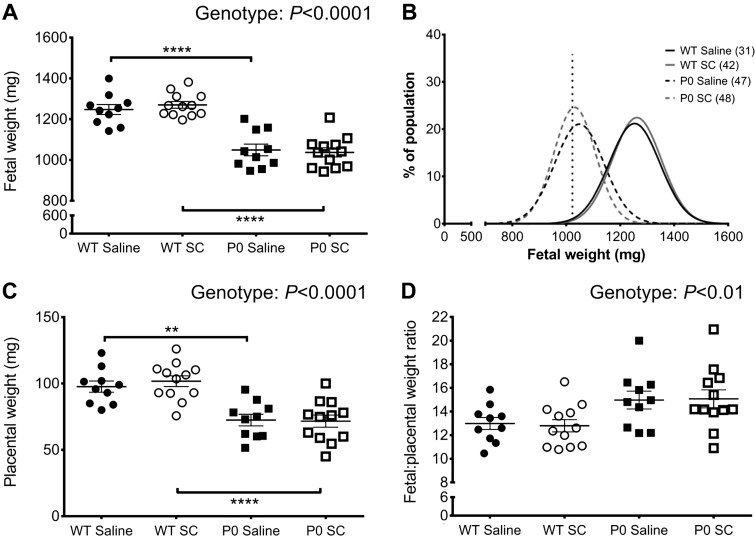
Fetal and placental weights at embryonic day (E)18.5 from wild-type (WT) and placental-specific insulin-like growth factor 2 knockout (P0) mice following subcutaneous sildenafil citrate (SC) 10 mg/kg or saline (SC *n* = 12 litters, saline *n* = 10 litters). *A*: fetal weight (litter averages by genotype). *B*: fetal weight frequency distribution curve of individual fetuses. Vertical gray dashed line denotes 5th centile of WT fetal weights (1.08 g), number of fetuses in parentheses. *C*: placental weight. *D*: fetal/placental weight ratios. Genotype and treatment were analyzed by 2-way ANOVA with Bonferroni post hoc test. ****P < 0.0001, **P < 0.01.

The mean number of fetuses per litter was not significantly different between saline control and treated groups (8.3 ± 0.3 vs. 7.8 ± 0.5, respectively, *P* = 0.48). SC had no effect on the mean number of fetal resorptions per litter compared with saline control (0.4 ± 0.1 vs. 0.8 ± 0.3, respectively, *P* = 0.20). SC did not affect the WT/P0 ratio of fetuses in each litter. In accord with previous studies (7, 10, 24, 34), fetal weight was significantly reduced in P0 vs. WT mice (*P* < 0.0001; Fig. 2*A*). SC had no effect on WT or P0 fetal weight at E18.5 (Fig. 2*A*). A frequency distribution curve of fetal weights from saline control and SC-treated dams is shown in Fig. 2*B*. The 5th centile of saline control WT fetal weights was 1.08 g, with 70% of P0 littermates falling below this 5th centile value; 70% of P0 mice from SC-treated dams also fell below the 5th centile of saline control WT weight.

Consistent with previous findings, placental weight was significantly reduced in P0 mice vs. WT littermates (*P* < 0.001; Fig. 2*C*). SC did not affect placental weight in either WT or P0 mice (Fig. 2*C*). There was a significant increase in fetal-to-placental weight ratio in P0 fetuses compared with WT, independent of treatment (*P* < 0.01; Fig. 2*D*). However, post hoc analysis showed no further differences in fetal/placental weight ratio between individual groups.

### Postnatal Growth

**Fig. 3.**
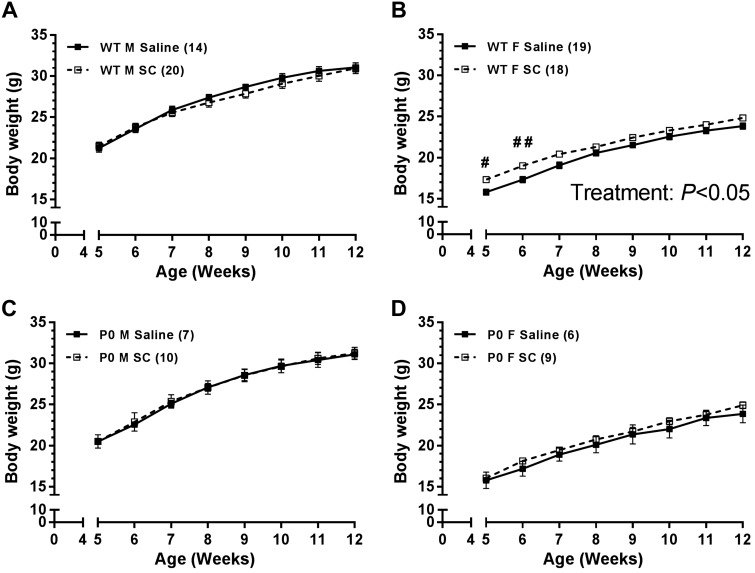
Offspring bodyweight from wild-type (WT; *A* and *B*) and placental-specific insulin-like growth factor 2 knockout (P0; *C* and *D*) mice following subcutaneous sildenafil citrate (SC, 10 mg/kg) or saline in male (*A* and *C*) and female (*B* and *D*) offspring. A 2-way ANOVA with Bonferroni post hoc test was used to compare the effect of treatment on body weight between saline and SC groups. For post hoc test, #*P* < 0.05, ##*P* < 0.01. Data are means ± SE.

Body weights from offspring of SC-treated pregnancies were compared with offspring from saline-treated pregnancies (Fig. 3). SC treatment did not affect body weight of male offspring between 5 and 12 wk of age irrespective of genotype (Fig. 3, *A* and *C*). Female P0 offspring exposed to SC in utero were similar in body weight to female offspring from saline-treated pregnancies (Fig. 3*D*). However, WT female offspring from SC-treated pregnancies were significantly heavier than WT female offspring from saline-treated pregnancies (Fig. 3*B*, *P* < 0.05); this difference was significant at postnatal *week 5* (17.3 ± 0.3 vs. 15.8 ± 0.4 g, respectively) and *week 6* (19.0 ± 0.3 vs. 17.3 ± 0.4 g, respectively). There were no differences in body weight when glucose tolerance and SBP were first measured.

### Postnatal Glucose Tolerance

**Fig. 4.**
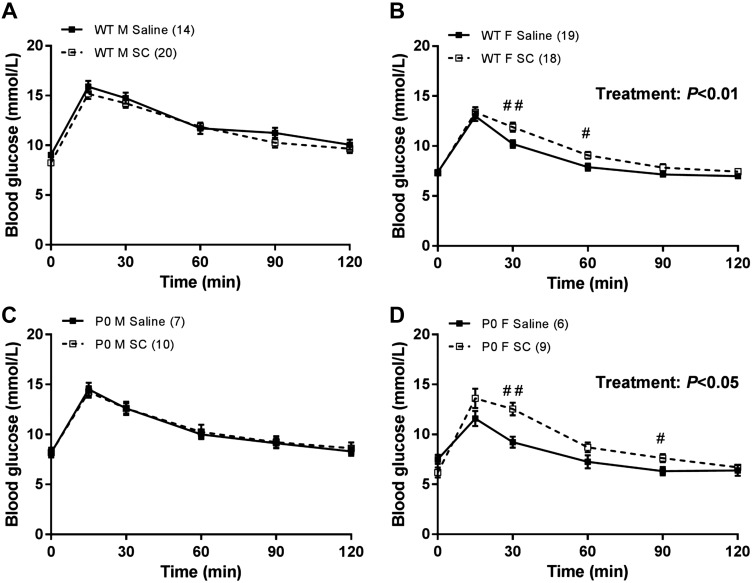
Offspring glucose tolerance from wild-type (WT; *A* and *B*) and placental-specific insulin-like growth factor 2 knockout (P0; *C* and *D*) mice following subcutaneous sildenafil citrate (SC, 10 mg/kg) or saline in male (*A* and *C*) and female (*B* and *D*) offspring. A Mann-Whitney *U*-test was used to assess differences in area under the curve (AUC) between saline and SC groups. At each individual time point, mean blood glucose concentrations from saline and SC offspring were also compared using a Mann-Whitney *U*-test. #*P* < 0.05, ##*P* < 0.01. Data are means ± SE.

Antenatal SC treatment had no effect on blood glucose concentration either before injection (fasting) or at any time point post-glucose challenge in male offspring compared with offspring of saline-treated dams irrespective of genotype (Fig. 4, *A* and *C*). Conversely, antenatal SC treatment significantly increased blood glucose concentrations in female offspring from both WT (saline AUC: 1,036 ± 28.6, SC AUC: 1,142 ± 24.5, means ± SE) and P0 (saline AUC: 940 ± 36.9, SC AUC: 1,123 ± 55.5) genotypes (*P* < 0.01 and *P* < 0.05, respectively; Fig. 4, *B* and *D*) compared with control offspring from saline-treated dams. In WT female offspring, this increase was most significant at *t* = 30 min (saline: 10.2 ± 0.4, SC: 11.9 ± 0.5 mmol/L, *P* < 0.01) and *t* = 60 min (saline: 8.0 ± 0.4, SC: 9.1 ± 0.3, *P* < 0.05), but there was no difference in fasting blood glucose concentration (Fig. 4*B*). In P0 female offspring, this increase was most significant at *t* = 30 min (saline: 9.2 ± 0.6, SC: 12.5 ± 0.6, *P* < 0.01) and *t* = 90 min (saline: 6.3 ± 0.4, SC: 7.6 ± 0.4, *P* < 0.05), but again there were no differences in fasting blood glucose concentration (*t* = 0 min; Fig. 4*D*).

### Postnatal SBP

**Fig. 5.**
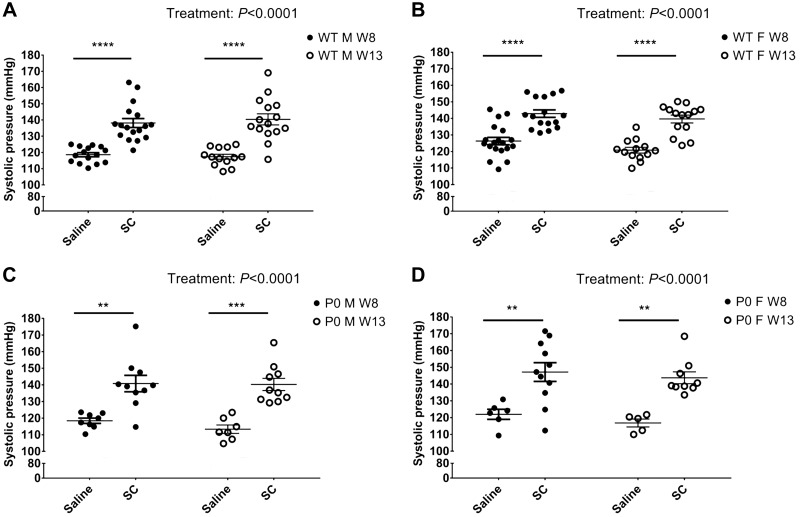
Systolic blood pressure (SBP) measurements from offspring from saline-treated and sildenafil citrate (SC)-treated pregnancies at 8 and 13 wk of age: wild-type (WT) males (*A*), WT females (*B*), placental-specific insulin-like growth factor 2 knockout (P0) males (*C*), and P0 females (*D*). Two-way ANOVA with Bonferroni post hoc test was used to compare effect of treatment and age on SBP. Data are means ± SE; each circle represents an individual animal. For post hoc test, **P < 0.01, ***P < 0.001, ****P < 0.0001.

At 8 and 13 wk of age, SBP was not different when the effect of genotype on offspring from saline-treated pregnancies was compared (Fig. 5).

Independently of sex or genotype, maternal administration of SC led to a significant increase in SBP in 8- and 13-wk-old offspring compared with offspring from saline-treated pregnancies (*P* < 0.01−*P* < 0.0001; Fig. 5, *A*–*D*).

### Postnatal Mesenteric Artery Vascular Function

**Fig. 6.**
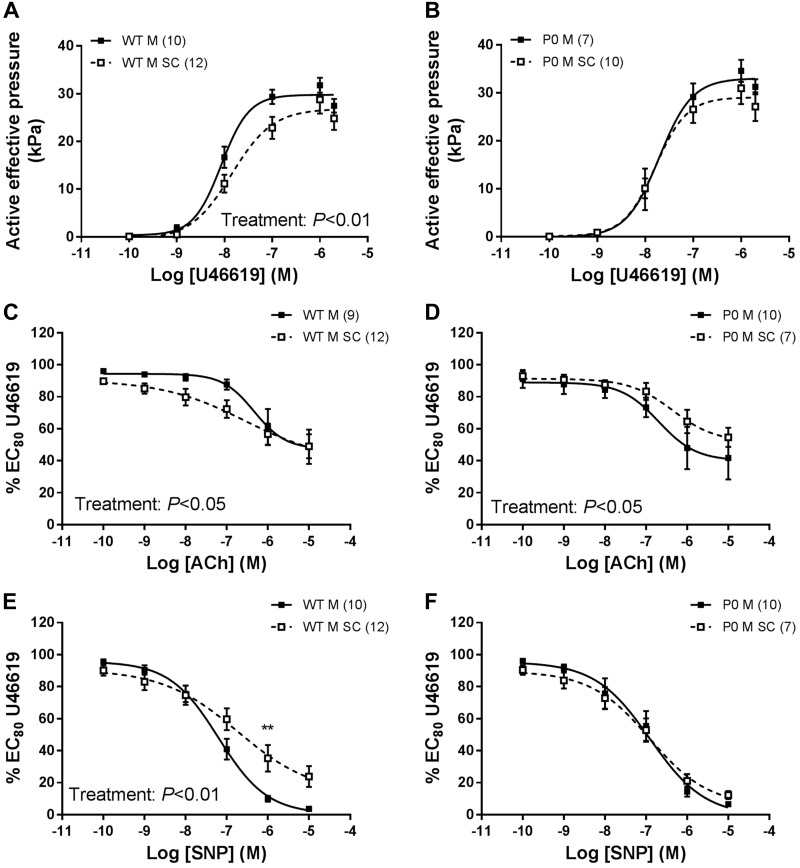
Dose-response curves of mesenteric arteries of male offspring from saline-treated and sildenafil citrate (SC)-treated pregnancies. *A* and *B*: mesenteric artery contraction in response to increasing doses of U46619 in wild-type (WT; *left*) and placental-specific insulin-like growth factor 2 knockout (P0; *right*) mice. *C* and *D*: relaxation of mesenteric arteries in response to ACh. *E* and *F*: relaxation of mesenteric arteries in response to SNP. Data are presented as means ± SE; number of offspring per group in parenthesis. Data were compared using 2-way ANOVA for effect of antenatal treatment with Bonferroni post hoc test. ACh, acetylcholine; SNP, sodium nitroprusside. For post hoc test, **P < 0.01.

There was a significant reduction in U46619-induced contraction of WT male offspring mesenteric arteries from SC-treated pregnancies compared with WT male offspring from saline-treated dams (Fig. 6*A*, *P* < 0.01), but this effect was not observed in P0 male offspring (Fig. 6*B*). There was an increased ACh-induced relaxation of WT male mesenteric arteries in offspring from SC-treated dams (Fig. 6*C*, *P* < 0.05) compared with saline-treated dams. Conversely, SC led to a reduced ACh-induced relaxation of mesenteric arteries from P0 males compared with P0 offspring from saline-treated pregnancies (*P* < 0.05; Fig. 6*D*). SNP-induced relaxation of mesenteric arteries from WT male offspring was reduced from SC-treated pregnancies compared with equivalent saline controls (*P* < 0.01; Fig. 6*E*). In contrast, SC during pregnancy did not alter relaxation responses of male offspring mesenteric arteries to SNP in P0 mice (Fig. 6*F*).

**Fig. 7.**
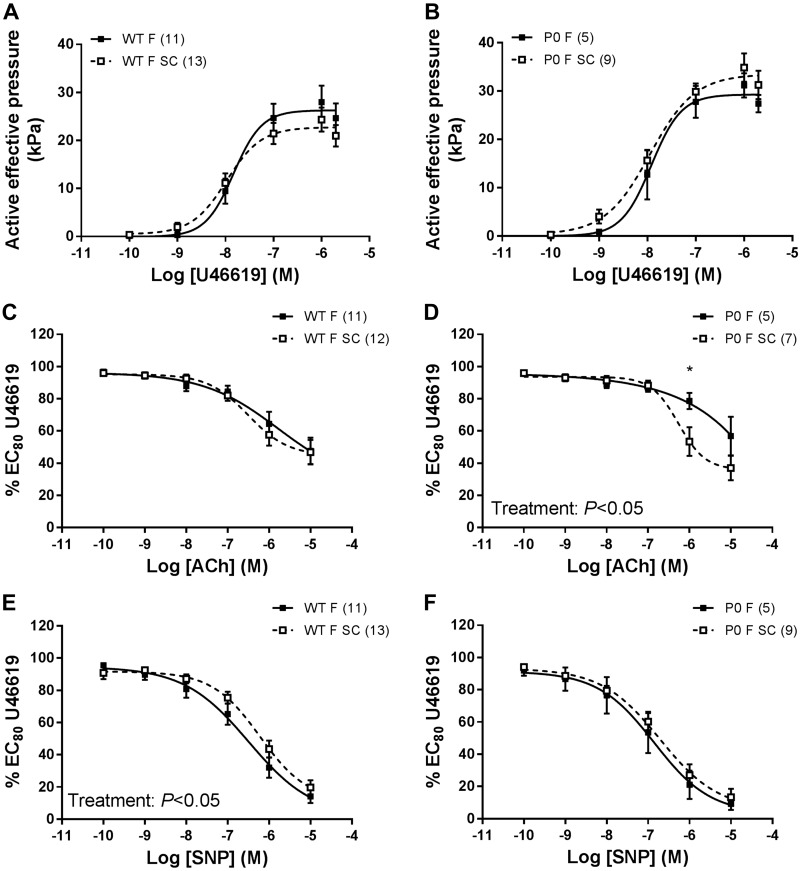
Dose-response curves of mesenteric arteries of female offspring from saline-treated and sildenafil citrate (SC)-treated pregnancies. *A* and *B*: mesenteric artery contraction in response to increasing doses of U46619 in wild-type (WT; *left*) and placental-specific insulin-like growth factor 2 knockout (P0; *right*) mice. *C* and *D*: relaxation of mesenteric arteries in response to ACh. *E* and *F*: relaxation of mesenteric arteries in response to SNP. Data are presented as means ± SE; number of offspring per group in parenthesis. Data were compared using 2-way ANOVA for effect of antenatal treatment with Bonferroni post hoc test. ACh, acetylcholine; SNP, sodium nitroprusside. For post hoc test, *P < 0.05.

SC treatment did not alter U46619-induced contraction of mesenteric arteries from WT or P0 female offspring (Fig. 7, *A* and *B*). ACh-induced relaxation was increased in P0 female mesenteric arteries in offspring from SC-treated vs. saline controls (*P* < 0.05; Fig. 7*D*). There was a significant reduction in SNP-induced relaxation of offspring mesenteric arteries in WT female mice from SC-treated pregnancies compared with saline controls (*P* < 0.05; Fig. 7*E*) but no difference in P0 female offspring (Fig. 7*F*).

### Offspring Organ Allometry

There was no effect of antenatal SC on weights of the heart, spleen, kidneys, or brain compared with weights of offspring from saline-treated pregnancies. However, lung weight of P0 male offspring was reduced by antenatal SC treatment vs. saline treatment (*P* < 0.05; Table 1). Conversely, heart weight of P0 male offspring was increased by antental SC treatment vs. saline treatment (*P* < 0.05; Table 1).

**Table 1.** Offspring organ allometry from saline- and sildenafil citrate-treated pregnancies

	Heart Weight		Average Kidney Weight		Spleen Weight		Lung Weight		Brain Weight	
Offspring ID	Value	*n*	Value	*n*	Value	*n*	Value	*n*	Value	*n*
Saline										
Male										
WT	0.51 [0.41–0.62]	11	1.16 [1.03–1.36]**	11	0.23 [0.20–0.29]**	11	0.46 [0.43–0.54]***	10	1.28 [1.19–1.56]****	11
P0	0.43 [0.39–0.66]*	7	1.15 [1.03–1.29]	7	0.23 [0.19–0.27]*	7	0.46 [0.44–0.58]*	7	1.31 [1.20–1.40]	6
Female										
WT	0.51 [0.42–0.80]	18	1.04 [0.94–1.20]**	18	0.31 [0.20–0.37]**	18	0.57 [0.52–0.69]***	16	1.73 [1.54–2.00]****	18
P0	0.51 [0.46–0.52]	4	1.01 [0.97–1.21]	5	0.35 [0.26–0.37]*	4	0.54 [0.47–0.57]	3	1.61 [1.51–1.88]	4
Sildenafil citrate (10 mg/kg)										
Male										
WT	0.49 [0.38–0.71]	19	1.11 [0.97–1.38]	16	0.23 [0.20–0.30]	16	0.52 [0.41–0.63]	16	1.39 [1.22–1.66]	16
P0	0.51 [0.43–0.72]*	9	1.17 [1.01–1.23]	6	0.23 [0.21–0.28]	6	0.43 [0.39–0.56]*	6	1.33 [1.26–1.43]	6
Female										
WT	0.47 [0.37–0.67]	16	1.05 [0.90–1.34]	15	0.33 [0.29–0.38]	15	0.54 [0.44–0.70]	15	1.65 [1.34–1.83]	15
P0	0.47 [0.44–0.63]	7	0.97 [0.87–1.05]	4	0.32 [0.31–0.36]	4	0.53 [0.44–0.54]	4	1.61 [1.41–1.80]	4

Values are medians [min–max] from 11 litters. P0, placental-specific insulin-like growth factor 2 knockout; WT, wild type. Organ wet weights measured at *week 14* and presented as %body weight. Organ weights were assessed for effect of genotype and sex within saline-treated groups by using the Kruskal-Wallis statistical test with identical letters denoting significance between groups using Dunn’s multiple comparisons post hoc test. A Mann-Whitney *U*-test was used to assess the effect of treatment between two groups; e.g., WT males from saline-treated pregnancy vs. WT males from sildenafil citrate-treated pregnancy.

* *P* < 0.05,

** *P* < 0.01,

*** *P* < 0.001, and

**** *P* < 0.0001 for post hoc test.

## DISCUSSION

There are currently no treatments for FGR. SC has been considered a promising therapeutic because of its beneficial effects on fetal growth in a number of animal models of FGR. However, recent randomized clinical trials have demonstrated no effect of SC on fetal growth in cases of severe early onset FGR (18, 41); effects on the offspring of these pregnancies remain unknown. In this study, we have demonstrated, contrary to our hypothesis, that exposure to SC increases SBP and impairs glucose tolerance in adult mouse offspring. Additionally, we have shown that subcutaneous administration of SC has no effect on fetal growth.

### Litter Characteristics

In the *COMT*^−/−^ and P0 mouse models of FGR, SC treatment, delivered via drinking water, led to an increase in fetal weight (10, 43) and, in the *COMT*^−/−^ mouse, normalized abnormal umbilical artery Doppler waveforms (43). In the present study, we observed a growth-restricted phenotype consistent with our previous reports on the P0 mouse (7, 10, 12, 24, 34). Also in keeping with our previous reports (10), there were no effects of SC on litter size or number of resorptions in P0 mice. However, in contrast to the study of Dilworth et al. (10), where SC was given via drinking water, subcutaneous administration of SC to P0 dams at 10 mg/kg daily (E12.5–E17.5) in the present study did not increase fetal or placental weight.

The disparity between results from within our group in the effectiveness of SC to increase fetal weight in the P0 mouse would seem to be a consequence of dosing regimen. Unlike in our earlier study (10), we chose to use a subcutaneous injection of SC as a means to standardize the therapeutic concentration of SC across dams. Furthermore, our dosing regimen equates to a human equivalent dose (29) of 56.7 mg SC per day (based on a pregnancy weight of 70 kg), which is within the range of, and no higher than, dosages used in human clinical trials (16, 18, 35, 41). By use of this approach, maternal plasma showed a peak SC and d-SC concentration (693 and 27 ng/mL, respectively) 1 h postinjection. Both SC and d-SC were still present 24 h postinjection but had reduced significantly. Fetal plasma showed a peak SC and d-SC concentration (27 and 6 ng/mL, respectively) 1 h postinjection, and both were still measurable at 24 h postinjection but had declined significantly. Importantly, maternal and fetal SC concentrations were well within maximum tolerated doses (46). Both SC and d-SC have been detected in rat fetal liver after maternal oral administration of SC (31), but this is the first study to directly measure SC and d-SC in mouse fetal plasma and as such the first to confirm SC transfer across the mouse placenta. Previous preclinical studies showing beneficial effects of SC on fetal weight had given mice ad libitum access to SC in water, whereas our study involved a single daily dose of SC via subcutaneous injection. The doses used in previous studies equate to a human equivalent dose of ~228 mg (43) and 456 mg (10) of SC per day and are therefore higher than the dosage used in our study. Such differences in dosing regimen may underlie the differential efficacy of SC to increase fetal growth.

### Postnatal Weight

Although it was not the primary focus of our study, we demonstrated that P0 mice, irrespective of sex, have similar growth trajectories from postnatal *week 5* compared with WT littermates. A recent publication by Mikaelsson et al. (26) demonstrated that P0 mice weighed 25% less than WT littermates at birth but demonstrated accelerated growth between 25 and 50 days of age, resulting in P0 mice having a near-normal, but still 7% lighter, body weight at postnatal *day 100*. However, Mikaelsson et al. did not assess the effect of sex on postnatal growth; thus, comparisons between the studies can be made only among male mice. It is also important to note that Mikaelsson et al. utilized the CD1 genetic strain of mouse, which have a larger litter size resulting in reduction in birth weight and increased competition during the lactation period compared with the C57BL/6J background strain used in this study.

Male mice exposed to SC in utero had similar body weights to their equivalent WT or P0 saline controls. However, WT female offspring exposed to SC in utero had significantly increased body weights at 5 and 6 wk of age compared with saline controls; this effect was not observed in P0 female offspring. The mechanisms for increased body weight in WT female mice from SC-treated dams are unknown.

### Glucose Tolerance

Blood glucose concentration was significantly higher in both WT and P0 female offspring from SC-treated dams compared with corresponding saline controls. Unlike studies purporting to show that females are protected against metabolic syndrome in cases of high-fat diet (32), treatment of dams with SC selectively reduced glucose sensitivity in female but not male offspring. Combined with the effect of antenatal SC on female offspring growth, these data suggest that female mice are more susceptible to the effects of antenatal SC. A recent study suggests that exposure to SC in utero potentiates the impaired glucose tolerance and increases insulin resistance in male C57BL/6J offspring at postnatal *day 150*, when they are exposed to a secondary insult such as a high-fat diet (28). Our study and that of Mills et al. suggest that SC in utero may predispose offspring from normal pregnancies to glucose intolerance in later life.

### Systolic Blood Pressure

FGR babies are at increased risk of hypertension in later life, which is often associated with metabolic syndrome (20). We are the first, to the best of our knowledge, to assess SBP in both male and female offspring of the P0 mouse. Previous studies have shown that male P0 offspring are growth restricted at birth, demonstrate early life catch-up growth, but continue to be 7% lighter than WT littermates by 100 days of age (26). Although P0 fetuses are growth restricted near term, we saw no differences in SBP at 8 or 13 wk of age when we compared P0 and WT offspring from saline-treated pregnancies. A life course assessment of SBP may identify differences in blood pressure between WT and P0 offspring at later postnatal ages.

Exposure to SC in utero significantly increased SBP at both 8 and 13 wk of age in male and female offspring from growth-restricted and normal pregnancies. There was an average increase in SBP of ~24 mmHg in mice exposed to SC in utero. Our data are in accord with that of Mills et al. (28), who demonstrated that *eNOS*^−/−^ offspring exposed to SC in utero had increased SBP in later life. In contrast, this same study saw no increase in SBP in WT offspring after antenatal exposure to SC, different from our findings. The disparity between the effects of SC exposure in utero in control (WT) mice may be a consequence of the differences in dosing regimen or may simply be due to the age at which blood pressure was measured i.e., 13 wk vs. 20 wk.

### Mesenteric Artery Function

One potential mediator of increased SBP is increased peripheral vascular resistance, which might be caused by aberrant vascular function of resistance blood vessels (5, 40). Such dysfunction can be defined as an enhanced response to contractile agents and/or reduced endothelial-dependent and -independent relaxation (3, 37). However, there was no consistent dysfunction in contraction or relaxation responses that could underlie the increase in SBP in either WT or P0 offspring of SC-treated dams. This is in contrast to a study where changes in SBP in offspring following exposure to SC in pregnant *eNOS*^−/−^ mice were associated with an increase in U46619-induced contraction of mesenteric arteries of male mice at 21 wk of age (28).

### Conclusions

We have, for the first time, demonstrated that exposure to SC in utero is associated with raised SBP in offspring independent of sex or genotype. Although we acknowledge the clinical importance of these data, there are a number of limitations which should be addressed in future studies. We did not assess cardiovascular function or perform terminal cardiopulmonary analyses in offspring, which could have provided a mechanism for raised SBP associated with SC. Furthermore, we were unable to assess mechanisms associated with differences in body weight in female offspring that were exposed to SC in utero. The translational impact of our work could be improved with these data, and any associations with the STRIDER trial must therefore take into account these limitations.

There have been a number of experimental and clinical studies assessing the effects of antenatal SC treatment during pregnancy. A systematic review of experimental and clinical studies of SC for FGR (44) highlighted only one study where maternal administration of SC had detrimental effects on fetal well-being [a decrease in fetal weight (33)]. However, a later study in the SUAL sheep model of FGR revealed that SC led to reduced uterine artery blood flow in both SUAL and control ewes associated with hypotension, tachycardia, and reduced Po_2_ in fetuses from both th control and the SUAL ewes (27). The focus of previous studies has been to assess SC effects on the mother, fetus, and in some cases neonate, but only one study (28) has examined the effects of antenatal SC in the long term.

The findings of this study must be placed into the context of the recently published STRIDER clinical trials (18, 41) assessing the impact of SC in severe early onset FGR. There were no effects of SC treatment on the prolongation of pregnancy, birth weight, or rates of fetal death in two cohorts of women with dismal prognosis (18, 41). There are, however, potentially concerning indications that SC led to reductions in fetal ductus venosus blood flow (41), and recently, 11 neonatal deaths led to the cessation of the Dutch STRIDER trial (19). Neonatal follow-up (<2 yr) will be assessed in children from SC-treated pregnancies; however, the data provided herein and elsewhere (28) suggest that it is very important to continue follow-up into at least early adulthood.

In the present study, and in line with the published outcomes of the various STRIDER trials, we have demonstrated that there was no effect of SC on fetal weight or fetal viability near term. We have also demonstrated that maternal SC treatment elevated SBP in the offspring of these pregnancies as early as 8 wk of age in the mouse, irrespective of genotype or sex, through as yet unidentified mechanisms. Combined, the outcomes of our study and that of Mills et al. (28), and in line with the findings of the STRIDER consortium (17, 18, 41), do not support maternal SC treatment as a safe or effective treatment for human FGR.

This study highlights the importance of assessing both the short and long-term consequences of therapeutics administered during pregnancy. Such data from pre-clinical models are crucial in affording a more informed choice for the obstetrician and patient on the potential short- and long-term risk vs. benefits of treatment in utero.

## GRANTS

This study was supported by The Medical Research Council Program (MRC) Grant G0802770. M. R. Dilworth is supported by an MRC Career Development Award Grant MR/K024442/1.

## DISCLOSURES

No conflicts of interest, financial or otherwise, are declared by the authors.

## AUTHOR CONTRIBUTIONS

L.J.R., E.C.C., C.P.S., P.N.B., S.L.G., M.W., and M.R.D. conceived and designed research; L.J.R., E.C., E.T., M.W., and M.R.D. performed experiments; L.J.R. and E.T. analyzed data; L.J.R. interpreted results of experiments; L.J.R. prepared figures; L.J.R., E.T., M.W., and M.R.D. drafted manuscript; L.J.R., E.C.C., C.P.S., E.T., S.L.G., M.W., and M.R.D. edited and revised manuscript; L.J.R., E.C.C., E.C., C.P.S., P.N.B., E.T., S.L.G., M.W., and M.R.D. approved final version of manuscript.

## References

[1] Ashworth A Effects of intrauterine growth retardation on mortality and morbidity in infants and young children. Eur J Clin Nutr 52, Suppl 1: S34–S41, 1998. 9511018

[2] Barker DJ, Eriksson JG, Forsén T, Osmond C Fetal origins of adult disease: strength of effects and biological basis. Int J Epidemiol 31: 1235–1239, 2002. doi:10.1093/ije/31.6.1235. 12540728

[3] Brawley L, Itoh S, Torrens C, Barker A, Bertram C, Poston L, Hanson M Dietary protein restriction in pregnancy induces hypertension and vascular defects in rat male offspring. Pediatr Res 54: 83–90, 2003. doi:10.1203/01.PDR.0000065731.00639.02. 12646717

[4] Bukowski R, Hansen NI, Willinger M, Reddy UM, Parker CB, Pinar H, Silver RM, Dudley DJ, Stoll BJ, Saade GR, Koch MA, Rowland Hogue CJ, Varner MW, Conway DL, Coustan D, Goldenberg RL; Eunice Kennedy Shriver National Institute of Child Health and Human Development Stillbirth Collaborative Research Network Fetal growth and risk of stillbirth: a population-based case-control study. PLoS Med 11: e1001633, 2014. doi:10.1371/journal.pmed.1001633. 24755550 PMC3995658

[5] Christensen KL, Mulvany MJ Mesenteric arcade arteries contribute substantially to vascular resistance in conscious rats. J Vasc Res 30: 73–79, 1993. doi:10.1159/000158978. 8504198

[6] Constância M, Dean W, Lopes S, Moore T, Kelsey G, Reik W Deletion of a silencer element in Igf2 results in loss of imprinting independent of H19. Nat Genet 26: 203–206, 2000. doi:10.1038/79930. 11017078

[7] Constância M, Hemberger M, Hughes J, Dean W, Ferguson-Smith A, Fundele R, Stewart F, Kelsey G, Fowden A, Sibley C, Reik W Placental-specific IGF-II is a major modulator of placental and fetal growth Nature 417: 945–948, 2002. doi:10.1038/nature00819. 12087403

[8] Cottrell EC, Sibley CP From pre-clinical studies to clinical trials: Generation of novel therapies for pregnancy complications. Int J Mol Sci 16: 12907–12924, 2015. doi:10.3390/ijms160612907. 26062129 PMC4490478

[9] de Jong F, Monuteaux MC, van Elburg RM, Gillman MW, Belfort MB Systematic review and meta-analysis of preterm birth and later systolic blood pressure. Hypertension 59: 226–234, 2012. doi:10.1161/HYPERTENSIONAHA.111.181784. 22158643 PMC3266458

[10] Dilworth MR, Andersson I, Renshall LJ, Cowley E, Baker P, Greenwood S, Sibley CP, Wareing M Sildenafil citrate increases fetal weight in a mouse model of fetal growth restriction with a normal vascular phenotype. PLoS One 8: e77748, 2013. doi:10.1371/journal.pone.0077748. 24204949 PMC3813774

[11] Dilworth MR, Kusinski LC, Baker BC, Renshall LJ, Greenwood SL, Sibley CP, Wareing M Defining fetal growth restriction in mice: a standardized and clinically relevant approach. Placenta 32: 914–916, 2011. doi:10.1016/j.placenta.2011.08.007. 21889207

[12] Dilworth MR, Kusinski LC, Cowley E, Ward BS, Husain SM, Constância M, Sibley CP, Glazier JD Placental-specific Igf2 knockout mice exhibit hypocalcemia and adaptive changes in placental calcium transport. Proc Natl Acad Sci USA 107: 3894–3899, 2010. doi:10.1073/pnas.0911710107. 20133672 PMC2840526

[13] Figueras F, Gratacós E Update on the diagnosis and classification of fetal growth restriction and proposal of a stage-based management protocol. Fetal Diagn Ther 36: 86–98, 2014. doi:10.1159/000357592. 24457811

[14] Fisk NM, Atun R Market failure and the poverty of new drugs in maternal health. PLoS Med 5: e22, 2008. doi:10.1371/journal.pmed.0050022. 18215109 PMC2211556

[15] Flenady V, Koopmans L, Middleton P, Frøen JF, Smith GC, Gibbons K, Coory M, Gordon A, Ellwood D, McIntyre HD, Fretts R, Ezzati M Major risk factors for stillbirth in high-income countries: a systematic review and meta-analysis. Lancet 377: 1331–1340, 2011. doi:10.1016/S0140-6736(10)62233-7. 21496916

[16] Ganzevoort W, Alfirevic Z, von Dadelszen P, Kenny L, Papageorghiou A, van Wassenaer-Leemhuis A, Gluud C, Mol BW, Baker PN STRIDER: sildenafil therapy in dismal prognosis early-onset intrauterine growth restriction—a protocol for a systematic review with individual participant data and aggregate data meta-analysis and trial sequential analysis. Syst Rev 3: 23, 2014. doi:10.1186/2046-4053-3-23. 24618418 PMC3975177

[17] Groom KM, Ganzevoort W, Alfirevic Z, Lim K, Papageorghiou AT; STRIDER Consortium Clinicians should stop prescribing sildenafil for fetal growth restriction (FGR): comment from the STRIDER Consortium. Ultrasound Obstet Gynecol 52: 295–296, 2018. doi:10.1002/uog.19186. 30079989

[18] Groom KM, McCowan LM, Mackay LK, Lee AC, Gardener G, Unterscheider J, Sekar R, Dickinson JE, Muller P, Reid RA, Watson D, Welsh A, Marlow J, Walker SP, Hyett J, Morris J, Stone PR, Baker PN STRIDER NZAus: a multicentre randomised controlled trial of sildenafil therapy in early-onset fetal growth restriction. BJOG 126: 997–1006, 2019. doi:10.1111/1471-0528.15658. 30779295

[19] Hawkes N Trial of viagra for fetal growth restriction is halted after baby deaths. BMJ 362: k3247, 2018. doi:10.1136/bmj.k3247. 30045911

[20] Huxley RR, Shiell AW, Law CM The role of size at birth and postnatal catch-up growth in determining systolic blood pressure: a systematic review of the literature. J Hypertens 18: 815–831, 2000. doi:10.1097/00004872-200018070-00002. 10930178

[21] Keijzer-Veen MG, Dülger A, Dekker FW, Nauta J, van der Heijden BJ Very preterm birth is a risk factor for increased systolic blood pressure at a young adult age. Pediatr Nephrol 25: 509–516, 2010. doi:10.1007/s00467-009-1373-9. 20012998 PMC2810359

[22] Khashu M, Narayanan M, Bhargava S, Osiovich H Perinatal outcomes associated with preterm birth at 33 to 36 weeks’ gestation: a population-based cohort study. Pediatrics 123: 109–113, 2009. doi:10.1542/peds.2007-3743. 19117868

[23] Kilkenny C, Browne WJ, Cuthill IC, Emerson M, Altman DG Improving bioscience research reporting: the ARRIVE guidelines for reporting animal research. PLoS Biol 8: e1000412, 2010. doi:10.1371/journal.pbio.1000412. 20613859 PMC2893951

[24] Kusinski LC, Dilworth MR, Baker PN, Sibley CP, Wareing M, Glazier JD System A activity and vascular function in the placental-specific Igf2 knockout mouse. Placenta 32: 871–876, 2011. doi:10.1016/j.placenta.2011.07.086. 21851977

[25] Lemons JA, Bauer CR, Oh W, Korones SB, Papile L-A, Stoll BJ, Verter J, Temprosa M, Wright LL, Ehrenkranz RA, Fanaroff AA, Stark A, Carlo W, Tyson JE, Donovan EF, Shankaran S, Stevenson DK; NICHD Neonatal Research Network Very low birth weight outcomes of the National Institute of Child Health and Human Development Neonatal Research network, January 1995 through December 1996. Pediatrics 107: e1, 2001. doi:10.1542/peds.107.1.e1. 11134465

[26] Mikaelsson MA, Constância M, Dent CL, Wilkinson LS, Humby T Placental programming of anxiety in adulthood revealed by Igf2-null models. Nat Commun 4: 2311, 2013. doi:10.1038/ncomms3311. 23921428

[27] Miller SL, Loose JM, Jenkin G, Wallace EM The effects of sildenafil citrate (Viagra) on uterine blood flow and well being in the intrauterine growth-restricted fetus. Am J Obstet Gynecol 200: 102.e1–102.e7, 2009. doi:10.1016/j.ajog.2008.08.029. 18845296

[28] Mills V, Plows JF, Zhao H, Oyston C, Vickers MH, Baker PN, Stanley JL Effect of sildenafil citrate treatment in the eNOS knockout mouse model of fetal growth restriction on long-term cardiometabolic outcomes in male offspring. Pharmacol Res 137: 122–134, 2018. doi:10.1016/j.phrs.2018.09.023. 30292428

[29] Nair AB, Jacob S A simple practice guide for dose conversion between animals and human. J Basic Clin Pharm 7: 27–31, 2016. doi:10.4103/0976-0105.177703. 27057123 PMC4804402

[30] Oyston C, Stanley JL, Oliver MH, Bloomfield FH, Baker PN Maternal administration of sildenafil citrate alters fetal and placental growth and fetal-placental vascular resistance in the growth-restricted ovine fetus. Hypertension 68: 760–767, 2016. doi:10.1161/HYPERTENSIONAHA.116.07662. 27432857

[31] Pellicer B, Herraiz S, Cauli O, Rodrigo R, Asensi M, Cortijo J, Serra V, Morcillo E, Felipo V, Simón C, Pellicer A Haemodynamic effects of long-term administration of sildenafil in normotensive pregnant and non-pregnant rats. BJOG 118: 615–623, 2011. doi:10.1111/j.1471-0528.2010.02839.x. 21244618

[32] Pettersson US, Waldén TB, Carlsson P-O, Jansson L, Phillipson M Female mice are protected against high-fat diet induced metabolic syndrome and increase the regulatory T cell population in adipose tissue. PLoS One 7: e46057, 2012. doi:10.1371/journal.pone.0046057. 23049932 PMC3458106

[33] Refuerzo JS, Sokol RJ, Aranda JV, Hallak M, Hotra JW, Kruger M, Sorokin Y Sildenafil citrate and fetal outcome in pregnant rats. Fetal Diagn Ther 21: 259–263, 2006. doi:10.1159/000091352. 16601334

[34] Renshall LJ, Dilworth MR, Greenwood SL, Sibley CP, Wareing M In vitro assessment of mouse fetal abdominal aortic vascular function. Am J Physiol Regul Integr Comp Physiol 307: R746–R754, 2014. doi:10.1152/ajpregu.00058.2014. 25056105 PMC4166756

[35] Samangaya RA, Mires G, Shennan A, Skillern L, Howe D, McLeod A, Baker PN A randomised, double-blinded, placebo-controlled study of the phosphodiesterase type 5 inhibitor sildenafil for the treatment of preeclampsia. Hypertens Pregnancy 28: 369–382, 2009. doi:10.3109/10641950802601278. 19843000

[36] Sasser JM, Baylis C Effects of sildenafil on maternal hemodynamics and fetal growth in normal rat pregnancy. Am J Physiol Regul Integr Comp Physiol 298: R433–R438, 2010. doi:10.1152/ajpregu.00198.2009. 19955496 PMC2828177

[37] Sathishkumar K, Balakrishnan MP, Yallampalli C Enhanced mesenteric arterial responsiveness to angiotensin II is androgen receptor-dependent in prenatally protein-restricted adult female rat offspring. Biol Reprod 92: 55, 2015. doi:10.1095/biolreprod.114.126482. 25550341 PMC4342791

[38] Satterfield MC, Bazer FW, Spencer TE, Wu G Sildenafil citrate treatment enhances amino acid availability in the conceptus and fetal growth in an ovine model of intrauterine growth restriction. J Nutr 140: 251–258, 2010. doi:10.3945/jn.109.114678. 20018809

[39] Scaffidi J, Mol BW, Keelan JA The pregnant women as a drug orphan: a global survey of registered clinical trials of pharmacological interventions in pregnancy. BJOG 124: 132–140, 2017. doi:10.1111/1471-0528.14151. 27297096

[40] Schiffrin EL Reactivity of small blood vessels in hypertension: relation with structural changes. State of the art lecture. Hypertension 19, Suppl: II1–II9, 1992. doi:10.1161/01.HYP.19.2_Suppl.II1-a. 1735561

[41] Sharp A, Cornforth C, Jackson R, Harrold J, Turner MA, Kenny LC, Baker PN, Johnstone ED, Khalil A, von Dadelszen P, Papageorghiou AT, Alfirevic Z; STRIDER group Maternal sildenafil for severe fetal growth restriction (STRIDER): a multicentre, randomised, placebo-controlled, double-blind trial. Lancet Child Adolesc Health 2: 93–102, 2018. doi:10.1016/S2352-4642(17)30173-6. 30169244

[42] Sibley CP, Coan PM, Ferguson-Smith AC, Dean W, Hughes J, Smith P, Reik W, Burton GJ, Fowden AL, Constância M Placental-specific insulin-like growth factor 2 (Igf2) regulates the diffusional exchange characteristics of the mouse placenta. Proc Natl Acad Sci USA 101: 8204–8208, 2004. doi:10.1073/pnas.0402508101. 15150410 PMC419581

[43] Stanley JL, Andersson IJ, Poudel R, Rueda-Clausen CF, Sibley CP, Davidge ST, Baker PN Sildenafil citrate rescues fetal growth in the catechol-O-methyl transferase knockout mouse model. Hypertension 59: 1021–1028, 2012. doi:10.1161/HYPERTENSIONAHA.111.186270. 22392899

[44] Villanueva-García D, Mota-Rojas D, Hernández-González R, Sánchez-Aparicio P, Alonso-Spilsbury M, Trujillo-Ortega ME, Necoechea RR, Nava-Ocampo AA A systematic review of experimental and clinical studies of sildenafil citrate for intrauterine growth restriction and pre-term labour. J Obstet Gynaecol 27: 255–259, 2007. doi:10.1080/01443610701194978. 17464805

[45] von Dadelszen P, Dwinnell S, Magee LA, Carleton BC, Gruslin A, Lee B, Lim KI, Liston RM, Miller SP, Rurak D, Sherlock RL, Skoll MA, Wareing MM, Baker PN; Research into Advanced Fetal Diagnosis and Therapy (RAFT) Group Sildenafil citrate therapy for severe early-onset intrauterine growth restriction. BJOG 118: 624–628, 2011. doi:10.1111/j.1471-0528.2010.02879.x. 21392225

[46] Walker DK, Ackland MJ, James GC, Muirhead GJ, Rance DJ, Wastall P, Wright PA Pharmacokinetics and metabolism of sildenafil in mouse, rat, rabbit, dog and man. Xenobiotica 29: 297–310, 1999. doi:10.1080/004982599238687. 10219969

[47] Wareing M, Myers JE, O’Hara M, Baker PN Sildenafil citrate (Viagra) enhances vasodilatation in fetal growth restriction. J Clin Endocrinol Metab 90: 2550–2555, 2005. doi:10.1210/jc.2004-1831. 15713717

[48] Webb JF Canadian thalidomide experience. Can Med Assoc J 89: 987–992, 1963. 14076167 PMC1921912

[49] Witjes BC, Ahsman MJ, van der Nagel BC, Tibboel D, Mathot RA Simultaneous assay of sildenafil and desmethylsildenafil in neonatal plasma by ultra-performance liquid chromatography-tandem mass spectrometry. Biomed Chromatogr 24: 180–185, 2010. 19609867 10.1002/bmc.1268

